# Comparative analysis of solar pasteurization versus solar disinfection for the treatment of harvested rainwater

**DOI:** 10.1186/s12866-016-0909-y

**Published:** 2016-12-09

**Authors:** André Strauss, Penelope Heather Dobrowsky, Thando Ndlovu, Brandon Reyneke, Wesaal Khan

**Affiliations:** Department of Microbiology, Faculty of Science, Stellenbosch University, Private Bag X1, Stellenbosch, 7602 South Africa

**Keywords:** Solar Pasteurization, Solar Disinfection, Microbial Indicators, *Legionella* spp., *Pseudomonas* spp., EMA-qPCR

## Abstract

**Background:**

Numerous pathogens and opportunistic pathogens have been detected in harvested rainwater. Developing countries, in particular, require time- and cost-effective treatment strategies to improve the quality of this water source. The primary aim of the current study was thus to compare solar pasteurization (SOPAS; 70 to 79 °C; 80 to 89 °C; and ≥90 °C) to solar disinfection (SODIS; 6 and 8 hrs) for their efficiency in reducing the level of microbial contamination in harvested rainwater. The chemical quality (anions and cations) of the SOPAS and SODIS treated and untreated rainwater samples were also monitored.

**Results:**

While the anion concentrations in all the samples were within drinking water guidelines, the concentrations of lead (Pb) and nickel (Ni) exceeded the guidelines in all the SOPAS samples. Additionally, the iron (Fe) concentrations in both the SODIS 6 and 8 hr samples were above the drinking water guidelines. A >99% reduction in *Escherichia coli* and heterotrophic bacteria counts was then obtained in the SOPAS and SODIS samples. Ethidium monoazide bromide quantitative polymerase chain reaction (EMA-qPCR) analysis revealed a 94.70% reduction in viable *Legionella* copy numbers in the SOPAS samples, while SODIS after 6 and 8 hrs yielded a 50.60% and 75.22% decrease, respectively. Similarly, a 99.61% reduction in viable *Pseudomonas* copy numbers was observed after SOPAS treatment, while SODIS after 6 and 8 hrs yielded a 47.27% and 58.31% decrease, respectively.

**Conclusion:**

While both the SOPAS and SODIS systems reduced the indicator counts to below the detection limit, EMA-qPCR analysis indicated that SOPAS treatment yielded a 2- and 3-log reduction in viable *Legionella* and *Pseudomonas* copy numbers, respectively. Additionally, SODIS after 8 hrs yielded a 2-log and 1-log reduction in *Legionella* and *Pseudomonas* copy numbers, respectively and could be considered as an alternative, cost-effective treatment method for harvested rainwater.

## Background

Several countries around the world utilise alternative water sources, such as rainwater harvesting (RWH) and surface water, to meet the increasing water demand and augment available water supplies. Rainwater harvesting in particular has been identified by the South African government as an alternative and sustainable water source that could provide water directly to households [1, 2]. Rainwater is considered a pure water source, however, during the harvesting process, it can become polluted with microorganisms and atmospheric particles such as, organic and inorganic matter (e.g. heavy metals and dust) [2–4]. Depending on the roof maintenance, leaves, animal faecal matter (which may contain chemicals such as phosphorous, nitrogen and trace elements) [4] and other debris particles, may also wash into the rainwater storage tank after a rain event and negatively affect the microbial quality of the tank water [4–6].

It has thus been concluded that stored harvested rainwater is not suitable for potable purposes due to the microbial quality in particular not complying with drinking water standards as established by the Department of Water Affairs and Forestry (DWAF) [7] and World Health Organization (WHO) [8] and it was recommended that harvested rainwater should be treated before utilisation as a primary water source [5, 9]. In developing countries, particularly, researchers seek cost- and time-effective treatment methods in order to improve the quality of harvested rainwater, for utilisation as a potable water source and for other domestic activities [6]. Solar disinfection (SODIS) and solar pasteurization (SOPAS) systems have been considered as efficient and cost-effective treatment methods for harvested rainwater [1, 6].

A SODIS system is based on the effect of ultra-violet (UV) light and heat from the sun, which inactivates microorganisms [6, 10]. A very simple example of a SODIS system is outlined by Amin and Han [1] and Amin et al. [6] where a transparent container is filled with harvested rainwater, placed onto a reflective surface and is exposed to direct sunlight for at least 6 to 8 hrs. Advantages of this system include cost-effectiveness and due to its simplicity it can be implemented worldwide [11]. Recent studies have also shown that SODIS improves the microbial quality of harvested rainwater [1, 6], although certain microorganisms and endospores may persist. Furthermore, the turbidity of the water may decrease the efficiency of the system due to the systems’ dependence on direct UV radiation penetration. Although the SODIS system is easier to implement than the SOPAS system, the efficiency of both systems decreases with cloudy weather conditions [1, 6, 10] and both systems may not improve the chemical quality of the harvested rainwater [10, 12].

A SOPAS system relies on the thermal effect (at least 70 °C), without UV radiation to inactivate microbes [13]. An example of a simple SOPAS system is the contemporary solar geyser, where water fills the borosilicate glass tubes, which is exposed to solar radiation. The energy which is obtained from solar radiation is transferred to the water which effectively heats up [14]. In addition, the time needed to treat water will decrease with an increase in temperature. Thus, the time required to treat water will decrease with a factor of 10 for every 10 °C increase in temperature above 50 °C [15]. This system is considered a cost-effective treatment method that is not influenced by the turbidity of the water [16, 17]. Research has also indicated that microbes will be inactivated when the water reaches a temperature of 55 °C or higher [6, 18, 19]. In a study conducted by Dobrowsky et al. [17], an Apollo™ SOPAS system (manufactured in China) successfully reduced the bacterial indicator counts in the rainwater samples pasteurized at the temperature ranges of 72 to 74 °C, 78 to 81 °C, and 90 to 91 °C, to below the detection limit (≥99.9%). Furthermore, *Legionella* spp. and *Pseudomonas* spp. were detected at the higher pasteurization temperatures (>78 °C), using the Polymerase Chain Reaction (PCR), however the viability of these organisms at temperatures higher than 72 °C was not confirmed. In a follow up study, Reyneke et al. [20] then utilised ethidium monoazide bromide quantitative polymerase chain reaction (EMA-qPCR) to verify that viable *Legionella* spp. were detected in solar pasteurized rainwater samples (>70 °C).

Legionellosis is a lung infection caused by *Legionella* spp. where the bacterium enters the lungs by inhalation of aerosolized contaminated water. It is well known that *Legionella* can proliferate at high temperatures [17, 21], however the growth temperature for *Legionella* is between 25 °C and 45 °C with an optimum temperature of 36 °C [22]. In a recent study conducted by Reyneke et al. [20] the research group showed that *Legionella* spp. are viable at temperatures higher than 70 °C. Numerous *Pseudomonas* spp. are associated with water environments as well as heated water sources such as hot tubs, physiotherapy and hydrotherapy pools and whirlpools [23, 24]. This is one of the most common opportunistic pathogens associated with nosocomial infections in individuals with a vulnerable immune system [23]. It normally enters the human body through a skin wound or during surgery where it is then taken up into the blood stream leading to bacteraemia that could cause pneumonia, endocarditis, osteomyelitis, gastrointestinal infections, urinary tract infections and is a leading cause of septicaemia [24, 25]. *Pseudomonas* is generally spread through contaminated water that comes into contact with a human host, or surgical equipment and the hands of hospital personnel that transfer it to a patient in the case of nosocomial infections [23].

Results obtained by Dobrowsky et al. [17] and Reyneke et al. [20] however, also indicated that significant concentrations of iron (Fe), aluminium (Al), lead (Pb) and nickel (Ni) may have been leaching from the 100 L stainless steel storage tank of the Apollo™ SOPAS system, which may have negatively affected the chemical quality of the treated rainwater. In the current study a new Phungamanzi^TM^ SOPAS system, which was designed and manufactured in South Africa and which consists of a 125 L high grade polyethylene storage tank, was utilised for the solar pasteurization of rainwater. The primary aim of the current study was to conduct a comparative analysis of the new SOPAS system versus SODIS for the treatment of rainwater. The treatment times of the SODIS systems included 6 and 8 hrs, while the treated rainwater for the SOPAS system was collected at different temperature ranges (70 to 79 °C; 80 to 89 °C; and 90 °C and above). To monitor the general microbial quality of the rainwater, indicator bacterial counts, including, *Escherichia coli* (*E. coli*), enterococci and faecal coliforms as well as the heterotrophic plate count (HPC), were determined using culture based methods. Chemical analysis was also performed (monitoring the concentration of cations and anions) in order to determine whether the treatment methods utilised alter the chemical quality of the rainwater. Finally, the efficiency of the two treatment methods in reducing the level of viable *Legionella* spp. and *Pseudomonas* spp. in roof harvested rainwater was analysed utilising EMA-qPCR. Ethidium monoazide bromide is a nucleic acid binding dye that can be used to bind to the deoxyribonucleic acid (DNA) of cells (after photoactivation) with damaged and permeable membranes (non-viable cells). The binding of the dye to the DNA prevents PCR amplification of the DNA and thereby leads to a strong signal reduction during qPCR as only the DNA from intact (viable) cells will be amplified [20, 26].

## Methods

### Description of the sampling site

**Fig. 1:**
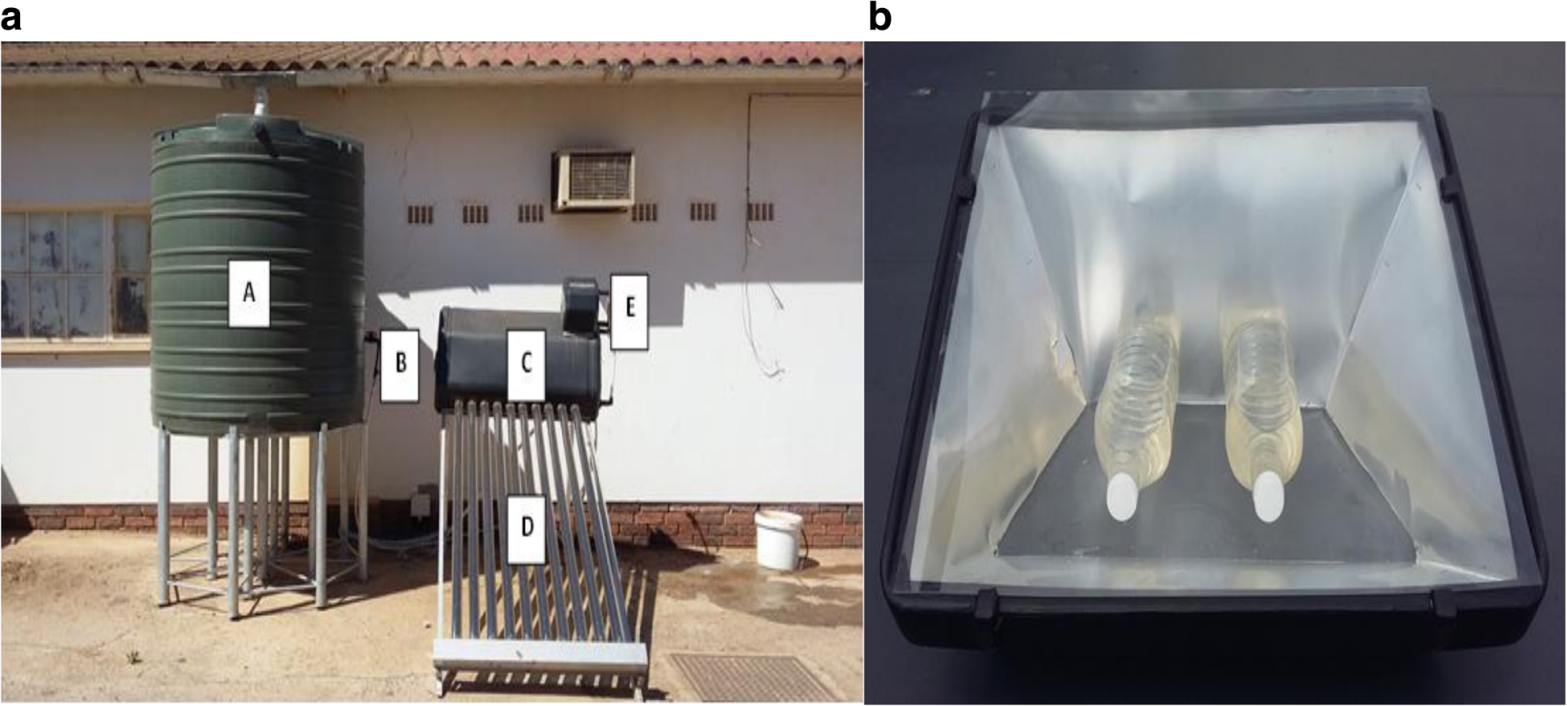
**a** The SOPAS system utilised in the current study was connected to a RWH tank installed on a metal stand. A: Untreated RWH tank (capacity: 2 000 L), B: Inlet pipe leading into the SOPAS tank, C: High grade polyethylene tank (capacity: 125 L), D: 10 × High borosilicate glass collector tubes, E: Outlet pipe and water collection point. **b** The SODIS system with two polyethylene terephthalate (PET) bottles containing harvested rainwater. The SODIS system was constructed from a black polyethylene material that was lined with a reflective aluminium surface. The system was covered with a transparent Perspex lid to increase insulation

A RWH system was installed on Welgevallen Experimental farm, Stellenbosch University (GPS co-ordinates: 33° 56′ 36.19″S, 18° 52′ 6.08″E), South Africa. The roof used as the catchment area was constructed from asbestos, while the gutter system leading to the polyethylene rainwater tank (2 000 L tank installed on a metal stand) was constructed from Chrysotile (white asbestos) (Fig. 1a). Furthermore, the sampling site is surrounded by trees and is located next to a dairy farm. However, no tree branches obstructed the catchment area.

#### Solar pasteurization system

The Phungamanzi^TM^ solar pasteurization system (manufactured in South Africa) was donated to Stellenbosch University by Crest Organization, Stellenbosch. This SOPAS system was connected to the 2 000 L polyethylene RWH tank, which was installed on a metal stand so that rainwater was able to flow from the rainwater storage tank into the SOPAS system in a passive manner (Fig. 1a). The water from the RWH tank flowed through the system components (Fig. 1a) as follows; water flowed from the RWH tank (A) through a pipe (B) into the high grade polyethylene tank (C) of the solar system, which has a 125 L storage capacity. The water then moved through the high borosilicate glass cylinders (D) in order to capture heat. Due to the thermo-siphoning effect, as the water was heated, the water moved into the main storage tank. The pasteurized water was then collected from the outlet tap (E).

#### Solar disinfection system

Two SUNSTOVE 2000™ solar oven systems (Sunstove Organization, South Africa), were placed on the rooftop of the JC Smuts building (33° 55′ 51.7″S 18° 51′ 55.3″E) at Stellenbosch University, South Africa, for the solar disinfection of the rainwater samples. As indicated in Fig. 1b, the solar oven has a very simplistic design, with the inside of the system constructed from a reflective aluminium plate and a black polyethylene material enclosing the system. In addition, in order to trap solar radiation, the inner section of the system was covered with a transparent Perspex lid.

### Sample collection

For both the SOPAS and SODIS systems, water samples were collected from July 2015 till October 2015, with a sampling event conducted one to four days after a rain event. Throughout the sampling period, for the SOPAS system, untreated rainwater (collected directly from RWH tank A) and solar pasteurized rainwater samples were collected in sterile 5 L polypropylene containers, respectively. Solar pasteurized samples were collected at the temperature ranges of 70 to 79 °C; 80 to 89 °C; and 90 °C and above. A MadgeTech TC101A thermocouple temperature Data Logger (MadgeTech, Inc.) was installed inside the SOPAS system in order to monitor the temperature of the treated rainwater for one month (01/08/2015 to 31/08/2015). The temperature data was obtained from the log tagger and analysed using the Data Logger Software Ver. 4.1.5 (Madge Tech, Inc.).

The SODIS treatment of rainwater was performed five times and for each sampling occasion, four sterile transparent 2 L polyethylene terephthalate (PET) bottles were filled to three-quarter capacity with roof harvested rainwater, obtained from the RWH tank A (Fig. 1a). Space was left in each bottle for aeration purposes and directly after collection each bottle was shaken for approximately 10 s in order to oxygenate the water [6, 27]. Two PET bottles were placed on the base of each respective SODIS system (Fig. 1b) and the one SODIS system was exposed to direct sunlight for 6 hrs, while the second SODIS system was exposed to direct sunlight for 8 hrs [28]. Furthermore, for each sampling occasion an untreated rainwater sample was also collected from tank A in a 5 L PET bottle.

The pH and temperature of each water sample was measured on site, using a hand-held pH meter (Milwaukee Instruments, Inc., USA) and mercury thermometer (ALLA® France, France), respectively. The daily temperature and rainfall data were obtained from the South African Weather Services (personal communication) and the solar irradiation data was obtained from the Stellenbosch Weather Services, Stellenbosch University, Faculty of Engineering (http://weather.sun.ac.za/).

### Chemical analysis

The chemical quality, including cation and anion concentrations of untreated and pasteurized (SOPAS) rainwater samples, collected for the various temperatures (cations: 71 °C, 86 °C and 93 °C) was determined. In addition, the chemical quality of untreated and SODIS rainwater samples collected after 6 hrs of treatment (cations: 70 °C and 89 °C) and 8 hrs of treatment (cations: 63 °C and 86 °C), were also analysed. For the determination of cation and metal ion concentrations, Falcon™ 50 mL high-clarity polypropylene tubes (Corning Life Sciences, USA) containing polyethylene caps were pre-treated with 1% nitric acid before sampling. The cation and metal ion concentrations [aluminium (Al), chromium (Cr), copper (Cu), iron (Fe), manganese (Mn), vanadium (V), and zinc (Zn), amongst others] were then determined using inductively coupled plasma atomic emission spectrometry (ICP-AES) [29]. This analysis was completed by the Central Analytical Facility (CAF), Stellenbosch University.

Furthermore, the anion analyses [SOPAS: untreated and 71 °C; SODIS untreated and treated at 6 hrs (52 °C; 70 °C and 89 °C) and 8 hrs (63 °C and 86 °C)] of the samples were performed by PathCare Reference Laboratory (PathCare Park, Cape Town, South Africa). All anions including, chloride, fluoride, nitrate and nitrite, phosphate and sulphates were measured utilising a Thermo Scientific Gallery™ Automated Photometric Analyser. The turbidity [​Nephelometric Turbidity Units (NTU)] of selected (untreated and treated) water samples was also determined by PathCare Reference Laboratory (PathCare Park, Cape Town, South Africa).

### Microbial analysis of treated and untreated rainwater samples

#### Enumeration of traditional indicator bacteria in rainwater samples

A serial dilution was prepared (10^−1^–10^−3^) for each rainwater sample collected during the sampling period [SOPAS (untreated and pasteurized samples) and SODIS (untreated and treated samples)] and using the spread plate method, 100 μL of the undiluted rainwater sample and each dilution (10^−1^ –10^−3^) was cultured in duplicate onto Slanetz and Bartley Agar (Oxoid, Hampshire, England) that was incubated for 44 - 48 hrs at 36 ± 2 °C, m-FC Agar (Merck, Darmstadt, Germany) that was incubated for 22 – 24 hrs at 35 ± 2 °C and R2A Agar (Oxoid, Hampshire, England) that was incubated for 72 – 96 hrs at 35 ± 2 °C, to enumerate enterococci, faecal coliforms and HPC, respectively.

For each sample, *E. coli* was enumerated by filtering a total volume of 100 mL (undiluted) through a sterile GN-6 Metricel® S-Pack Membrane Disc Filter (Pall Life Sciences, Michigan, USA) with a pore size of 0.45 μm and a diameter of 47 mm, at a filtration flow rate of approximately ≥ 65 mL/min/cm^2^ at 0.7 bar (70 kPa), in duplicate. The membrane filters were then incubated on Membrane Lactose Glucuronide Agar (MLGA) (Oxoid, Hampshire, England) at 35 ± 2 °C for 18 - 24 hrs.

#### Rainwater concentration, EMA treatment and DNA extraction

For each sampling event, 1 L rainwater sample [SOPAS (untreated and pasteurized samples) and SODIS (untreated and treated samples)] was concentrated as outlined in Reyneke et al. [20]. The concentrated rainwater samples utilised for *Legionella* spp. detection were treated with 2.5 μg/mL ethidium monoazide bromide (EMA) as previously described by Delgado-Viscogliosi et al. [30]. The same parameters were then utilised for the detection of *Pseudomonas* spp. in the concentrated rainwater samples. Following the addition of EMA, the samples were incubated on ice for 10 min followed by a 15 min halogen light exposure (keeping the samples on ice to avoid over-heating during the photoactivation step). The EMA treated samples were then washed with 1 mL NaCl (0.85%) followed by centrifugation (16 000 × g for 5 min). The DNA extractions were completed using the Soil Microbe DNA MiniPrep™ Kit (Zymo Research, USA) as per manufacturer’s instructions by first re-suspending the obtained pellet in the lysis solution and transferring the mixture to the ZR BashingBead™ Lysis Tubes.

#### Quantitative PCR for the detection of *Legionella* and *Pseudomonas* spp.

Following the EMA treatment and DNA extractions, EMA-qPCR was performed on a LightCycler®96 (Roche Applied Science, Mannheim, Germany) using the FastStart Essential DNA Green Master Mix (Roche Applied Science, Mannheim, Germany). To a final reaction volume of 20 μL, the following were added: 10 μL FastStart Essential DNA Green Master Mix (2x), 5 μL template DNA (diluted by 10 fold) and 0.4 μL of each primer (final concentration 200 nM) as previously described by Herpers et al. [31] for *Legionella* spp. and by Roosa et al. [32] for *Pseudomonas* spp.

For *Legionella* spp., the primers LegF (5′–CTAATTGGCTGATTGTCTTGAC–3′) and LegR (5′–CAATCGGAGTTCTTCGTG–3′) were utilised to amplify a 259 bp product of the 23S rRNA gene [31]. The amplification conditions for *Legionella* spp. were as follows: initial denaturation at 95 °C for 10 min, followed by 50 cycles of denaturation at 95 °C for 15 s, annealing at 60 °C for 15 s and extension at 72 °C for 11 s.

For *Pseudomonas* spp., the primers PS1 (5′-ATGAACAACGTTCTGAAATTC-3′) and PS2 (5′-CTGCGGCTGGCTTTTTCCAG-3′) were utilised to amplify a 249 bp product of the *Pseudomonas* lipoprotein *oprI* gene [33]. The amplification conditions for *Pseudomonas* spp. were as follows: initial denaturation at 95 °C for 10 min, followed by 50 cycles of denaturation at 94 °C for 30 s, annealing at 58 °C for 30 s and extension at 72 °C for 30 s.

The standard curves for the *Legionella* spp. qPCR assays were produced by amplifying the 23S rRNA gene of *Legionella pneumophila* ATCC 33152, using primers LegF and LegR. In addition, the standard curves for the *Pseudomonas* spp. qPCR assays were produced by amplifying the lipoprotein *oprI* gene of *P. aeruginosa* ATCC 27853, using primers PS1 and PS2. The PCR products were then purified using the DNA Clean & Concentrator™-5 Kit (Zymo Research) and were verified by DNA sequencing followed by quantifying the DNA in triplicate using the NanoDrop® ND-1000 (Nanodrop Technologies Inc., Wilmington, Delaware, USA). A serial 10-fold dilution (*Legionella* spp.: 10^8^ to 10^1^; *Pseudomonas* spp.: 10^9^ to 10^0^) of the PCR products was prepared in order to generate the standard curves, where the regression coefficient (*R* ^2^) was kept higher than 0.98 and 1.00 for *Legionella* and *Pseudomonas* spp., for each experiment, respectively. For *Legionella* spp. and *Pseudomonas* spp. detection, a concentration of 1.00 × 10^8^ and 1.00 × 10^9^ gene copies/μL was prepared for the dilution with the highest copy number, respectively, while a concentration of 1.00 × 10^1^ and 1.00 × 10^0^ gene copies/μL was prepared for the dilution with the lowest copy number. The standard curves were generated by plotting quantitative cycle (C_q_) values versus the log concentrations of standard DNA, as previously described by Chen and Chang [34], for determining the copy number of the 23S rRNA gene in *Legionella* spp. and the copy number of the lipoprotein *oprI* gene in *Pseudomonas* spp.in all samples analysed. Melt curve analysis was included for both *Legionella* and *Pseudomonas* spp. SYBR green real-time PCR assays in order to verify the specificity of the primer set by ramping the temperature from 65 to 97 °C at a rate of 0.2 °C/s with continuous fluorescent signal acquisition at 5 readings/°C.

### The determination of bacterial removal efficiency of the treatment systems

The bacterial removal efficiency of each treatment system (SOPAS and SODIS) was obtained by comparing the bacterial counts obtained from the samples collected before treatment and the average bacterial counts obtained from samples collected after treatment. The percentage reduction was calculated using Eq. 1 [35].

1 $$ \mathrm{Percentage}\ \mathrm{reduction} = 100\ \hbox{-} \left(\mathrm{Survivor}\ \mathrm{count}/\mathrm{Initial}\ \mathrm{count}\right) \times 100 $$

### Statistical analysis

The statistical software package Statistica™ Ver. 11.0 (Stat Soft Inc., Tulsa, USA) was used for the evaluation of the microbial analysis and the temperature of the collected rainwater samples (untreated, pasteurized and disinfected). To test the significance of the data set, an ANOVA analysis was performed for evenly distributed data while for non-evenly distributed data, a spearman rank order correlation was performed. A significant level of 5% was used as a standard in the hypothesis tests [36], while in all tests a *p*-value of <0.05 was considered statistically significant.

## Results

### Physico-chemical parameters for water samples collected from SOPAS and SODIS treatment systems

The temperature of the solar pasteurized water samples collected throughout the sampling period (n = 6) ranged from 71 °C (July 2015) to the highest temperature of 93 °C (October 2015). The temperature of the SODIS samples were also monitored after 6 hrs and 8 hrs of treatment, respectively, with the temperature of the 6 hr samples (n = 5) ranging from 52 °C (July 2015) to 89 °C (October 2015) and the temperature of the 8 hr SODIS samples (n = 5) ranging from 63 °C (August 2015) to 86 °C (October 2015). For both the SOPAS and the SODIS treatment, the highest total monthly rainfall over the sampling period was recorded in July 2015 (174.4 mm), which then decreased to 67.6 mm in August 2015, increased to 78.2 mm in September and then decreased to the lowest rainfall recorded in October 2015 (10.0 mm).

For the SODIS treatment, an overall average daily ambient temperature of 24.3 °C was recorded during the sampling period, with the lowest temperature of 17.2 °C recorded during July 2015 and the highest temperature of 29.7 °C recorded during October 2015. The temperature of the untreated water samples (collected directly from the RWH tank), averaged 20.2 °C, with the lowest temperature measured as 17.2 °C (July 2015) and the highest temperature measured as 25.2 °C (October 2015). In addition, an overall average pH of 8.0 was recorded for the untreated water samples, while an overall pH of 8.1 was recorded for the solar disinfected water samples after 6 hrs and 8 hrs of treatment, respectively.

For the SOPAS treatment, an overall average daily ambient temperature of 25.5 °C was recorded during the sampling period, with the lowest temperature of 17.2 °C recorded during July 2015 and the highest temperature of 30.6 °C recorded during October 2015. Similarly, the temperature of the untreated water samples (collected directly from the RWH tank), averaged 24.7 °C, with the lowest temperature measured as 19 °C (July 2015) and the highest temperature measured as 29.0 °C (October 2015). In addition, an overall average pH of 8.0 was recorded for the untreated water samples, while an overall pH of 7.6 was recorded for the solar pasteurized water samples.

Furthermore, a data logger probe was used to measure the water temperature inside the SOPAS system for a period of one month (01/08/2015 to 31/08/2015) (results not shown). An overall average ambient temperature of 21.1 °C was obtained with the lowest temperature recorded as 7.4 °C and the highest temperature recorded as 39.0 °C. In addition, the water temperature inside the SOPAS system had an overall average of 56.9 °C during the monitored month which ranged from 40.1 °C to 82.9 °C. Solar irradiation data was obtained from Stellenbosch Weather Service (Engineering Facility) and ranged from 0.01 W/m^2^ to 881.37 W/m^2^ with an overall average of 297.27 W/m^2^. A direct positive correlation between the ambient temperature and solar irradiation (R = 0.69; *p* < 0.05) and the temperature of the water inside the system (R = 0.20; *p* < 0.05) was also obtained.

### Chemical analysis of untreated and treated rainwater samples

#### Chemical analysis of the SOPAS rainwater samples

Untreated and solar pasteurized water samples (71 °C) collected during the first sampling event were analysed for their anion concentrations (results not shown). All anion concentrations of the untreated water sample and the solar pasteurized water sample were within the drinking water guidelines as stipulated by Australian Drinking Water Guidelines (ADWG) [37], DWAF [7] and South African National Standards (SANS) 241 [38]. A previous study conducted by Dobrowsky et al. [17] also indicated that there was no significant difference between the anion concentrations in the untreated and solar pasteurized water samples (55 to 91 °C). Anion analyses were thus not conducted on the untreated and solar pasteurized rainwater samples collected during the remainder of the sampling period. The turbidity of the untreated and pasteurized rainwater samples was also measured and according to DWAF [7], SANS 241 [38] and ADWG [37], the turbidity should not exceed 1.00 NTU. For both the untreated and solar pasteurized water sample, the turbidity was measured as 0.00 NTU, thus the turbidity complied with the respective drinking water guidelines.

The metal ions and cation concentrations were determined for pasteurized water samples collected at 71 °C, 86 °C and 93 °C and the corresponding unpasteurized samples (Table 1). The concentrations of the metal ions and cations in the untreated and SOPAS treated rainwater samples were below the recommended guidelines as stipulated by ADWG [37], DWAF [7] and SANS 241 [38], with the exception of Pb and Ni. However, while all the before and after SOPAS treatment samples were within the stipulated guidelines for Fe concentrations, the concentration of Fe in the before treatment sample (172.92 μg/L), collected with the corresponding 71 °C SOPAS sample, exceeded the DWAF [7] drinking water guideline of <100 μg/L. The Fe concentration in the SOPAS treatment sample collected at 71 °C then decreased significantly (*p* < 0.05) to 29.19 μg/L.

**Table 1:** Cation and metal ion concentrations of the untreated water samples and the corresponding solar pasteurized water samples collected at various temperatures compared to the recommended drinking water guidelines

Metal	Before 71 °C	After 71 °C	Before 86 °C	After 86 °C	Before 93 °C	After 93 °C	SANS 241	DWAF	AWDG
Al (μg/L)	1.98	99.18	1.36	37.99	1.13	31.70	300	150	200
B (μg/L)	< 0.1	37.77	-	-	-	-	-	-	4000
V (μg/L)	0.07	1.45	0.05	0.62	0.04	0.60	200	1000	-
Mn (μg/L)	4.78	8.60	1.09	9.94	2.16	9.48	100	50	500
Fe (μg/L)	172.92	29.19	78.91	50.07	51.55	26.89	200	100	300
Co (μg/L)	0.05	0.22	0.05	0.24	0.05	0.23	500	-	-
Ni (μg/L)	1.60	30.00	5.21	26.46	0.55	25.59	150	-	20
Cu (μg/L)	2.65	525.42	2.96	549.63	3.94	495.44	1000	1000	2000
Zn (μg/L)	26.45	2529.53	17.37	2086.09	6.70	2003.86	5000	3000	3000
As (μg/L)	0.25	5.63	0.37	1.58	0.49	1.48	10	10	10
Mo (μg/L)	0.02	0.26	0.01	0.15	0.02	0.14	-	-	50
Cd (μg/L)	< 0.05	0.49	0.00	0.77	0.00	0.58	5	5	2
Ba (μg/L)	29.86	86.30	92.97	78.62	88.75	73.53	-	-	2000
Pb (μg/L)	0.59	74.12	<0.006	26.30	<0.006	19.67	20	10	10
Ca (mg/L)	3.05	7.37	4.87	5.42	4.74	5.49	150	32	-
K (mg/L)	0.50	1.60	0.47	0.80	0.50	1.04	50	50	-
Mg (mg/L)	0.31	0.83	0.45	0.57	0.44	0.58	70	30	-
Na (mg/L)	1.61	3.67	2.07	2.70	2.09	2.70	200	100	180
P (mg/L)	0.04	0.08	0.04	0.05	0.03	0.04	-	-	-
Si (mg/L)	0.31	1.37	0.64	1.77	0.65	1.78	-	-	-

In addition, while Ni was within the SANS 241 [38] drinking water guideline in all the water samples analysed, it was detected above the drinking water guideline (<20 μg/L) according to ADWG [37] for all three samples collected after pasteurization (71 °C, 30.00 μg/L; 86 °C, 26.46 μg/L; and 93 °C, 25.59 μg/L). Furthermore, Pb was detected above the drinking water guideline stipulated by ADWG [37], DWAF [7] and SANS 241 [38] for all three samples collected after pasteurization (71 °C, 86 °C and 93 °C) with a concentration of 74.12 μg/L, 26.30 μg/L and 19.67 μg/L recorded, respectively. It should however, be noted that both the Ni and Pb concentrations decreased with an increase in SOPAS temperature.

#### Chemical analysis of the SODIS rainwater samples

All anion concentrations of the SODIS rainwater samples [untreated and treated at 6 hrs (anions: 52 °C; 70 °C and 89 °C) and 8 hrs (anions: 63 °C and 86 °C)], were within the drinking water guidelines as stipulated by ADWG [37], DWAF [7] and SANS 241 [38] (results not shown). In addition, there was no significant (*p* > 0.05) increase in the anion concentrations after treatment. While the turbidity measurements of all the water samples before and after treatment, were within the 1.00 NTU recommended guideline [7, 37, 38], the turbidity of samples collected during the first sampling event in August 2015, were not within the drinking water guidelines. It should however be noted that the untreated water sample had a turbidity of 1.90 NTU, which already exceed the drinking water guidelines. After 6 hrs of treatment by SODIS (70 °C), the turbidity increased to 2.14 NTU, while the sample treated for 8 hrs (63 °C) had a turbidity of 2.09 NTU.

The metal ions and cation concentrations were measured for representative SODIS sampling events [6 hrs (70 °C and 89 °C) and 8 hrs (63 °C and 86 °C) after treatment] and their corresponding untreated water sample (Table 2). Similar to the results obtained for the SOPAS treated water samples, the concentrations of all the metal ions and cations, in the untreated and SODIS rainwater samples were within the recommended guidelines as stipulated by ADWG [37], DWAF [7] and SANS 241 [38]. However, the concentrations of Fe in the untreated and treated (6 and 8 hrs) samples were significantly (*p* < 0.05) higher compared to the drinking water guidelines as stipulated by ADWG [37], DWAF [7] and SANS 241 [38]. The first untreated sample had an Fe concentration of 571.26 μg/L, which increased to 729.71 μg/L after 6 hrs of treatment (70 °C) and then decreased to a concentration of 645.39 μg/L after 8 hrs of treatment (63 °C). Similarly, an Fe concentration of 112.60 μg/L was recorded in the untreated sample corresponding to the temperature ranges of 89 °C (6 hrs of treatment) and 86 °C (8 hrs of treatment), with the Fe concentration increasing to 1015.32 μg/L (6 hrs) and decreasing to 505.35 μg/L after 8 hrs.

**Table 2:** Cation and metal ion concentrations of the untreated water samples and the corresponding SODIS treated water samples collected after 6 and 8 hrs compared to the recommended drinking water guidelines

Metal	Untreated	After 6 hrs (70 °C)	After 8 hrs (63 °C)	Untreated	After 6 hrs (89 °C)	After 8 hrs (86 °C)	SANS 241	DWAF	AWDG
Ti (μg/L)	0.51	0.53	0.32	0.09	0.12	0.13	**-**	**-**	**-**
Al (μg/L)	14.35	20.29	11.82	1.19	3.77	5.61	300	150	200
V (μg/L)	0.21	0.20	0.25	0.07	0.14	0.16	200	1000	-
Cr (μg/L)	0.06	0.04	0.06	0.12	0.12	0.13	100	50	50
Mn (μg/L)	3.31	3.24	3.09	1.09	15.30	13.59	100	50	500
Fe (μg/L)	571.26	729.71	645.39	112.60	1015.32	505.35	200	100	300
Co (μg/L)	0.03	0.04	0.03	0.05	0.14	0.13	500	-	-
Ni (μg/L)	0.26	0.25	0.15	0.54	0.46	0.44	150	-	20
Cu (μg/L)	3.09	2.35	9.72	1.00	1.10	1.50	1000	1000	2000
Zn (μg/L)	1.46	5.29	4.18	4.50	5.12	2.52	5000	3000	3000
As (μg/L)	0.45	0.54	0.50	0.38	0.64	0.52	10	10	10
Mo (μg/L)	<0.005	<0.005	<0.005	0.02	0.01	0.03	-	-	50
Cd (μg/L)	<0.004	<0.004	<0.004	0.00	0.00	0.00	5	5	2
Ba (μg/L)	3.79	3.75	2.93	98.05	99.33	89.68	-	-	2000
Pb (μg/L)	0.46	0.65	0.53	<0.006	0.10	0.16	20	10	10
Ca (mg/L)	2.86	2.84	2.88	4.83	4.83	4.83	150	32	-
K (mg/L)	0.35	0.38	0.40	0.49	0.49	0.47	50	50	-
Mg (mg/L)	0.35	0.36	0.37	0.45	0.44	0.46	70	30	-
Na (mg/L)	2.01	2.05	2.08	2.06	2.00	2.09	200	100	180
P (mg/L)	0.10	0.14	0.15	0.04	0.08	0.14	-	-	-

### Indicator bacterial counts in untreated and treated rainwater samples

#### Indicator bacteria detected in untreated and SOPAS rainwater samples

For each untreated water sample and the corresponding pasteurized sample collected at various temperatures ranging from 71 °C to 93 °C, water samples were analysed for the presence of indicator bacteria including *E. coli*, HPC, enterococci and faecal coliforms (Table 3). Enterococci and faecal coliforms were not detected in any of the untreated as well as the pasteurized rainwater samples. However, the HPC for the untreated water samples ranged from a minimum of 7.05 × 10^6^ CFU/100 mL to a maximum of 7.4 × 10^7^ CFU/100 mL and were reduced to below the detection limit (<1 CFU/mL) after pasteurization for all temperature ranges (71 °C to 93 °C).

**Table 3:** Indicator counts for solar pasteurized water samples and the corresponding untreated water samples collected at various temperatures

Pasteurization Temperature	Indicator	Untreated Water Sample (Ave. CFU/100 mL)	Treated Water Sample (Ave. CFU/100 mL)	Reduction (%)
71 °C	*E. coli*	2	BDL	>99
	HPC	7.05 × 10^6^	BDL	>99
77 °C	*E. coli*	2	BDL	>99
	HPC	6.62 × 10^7^	BDL	>99
81 °C	*E. coli*	2	BDL	>99
	HPC	1.01 × 10^7^	BDL	>99
86 °C	*E. coli*	2	BDL	>99
	HPC	1.46 × 10^7^	BDL	>99
91 °C	*E. coli*	2	BDL	>99
	HPC	1.43 × 10^7^	BDL	>99
93 °C	*E. coli*	3	BDL	>99
	HPC	7.4 × 10^7^	BDL	>99

(Note: *BDL* below detection limit)

*Escherichia coli* were also detected in all the untreated water samples with a minimum of 2 CFU/100 mL to a maximum of 3 CFU/100 mL recorded. Similarly, *E. coli* counts were reduced to below the detection limit after pasteurization (71 °C to 93 °C). For the untreated rainwater samples, both the HPC and the *E. coli* counts exceeded the drinking water guidelines as stipulated by the DWAF [7]. However, after pasteurization a >99% reduction in indicator counts was observed for all the pasteurized rainwater samples and the counts were within the DWAF [7] standards.

#### Indicator bacteria detected in untreated and SODIS rainwater samples

For each untreated water sample and the corresponding solar disinfected water sample, collected at various temperatures ranging from 52 °C to 89 °C and 63 °C to 86 °C treated for 6 hrs and 8 hrs, respectively, water samples were analysed for the presence of indicator bacteria including *E. coli*, HPC, enterococci and faecal coliforms. Similar to results obtained for the SOPAS samples, enterococci and faecal coliforms were not detected in any of the untreated as well as both the 6 hr and 8 hr disinfected water samples. However, the HPC in all the untreated water samples ranged from a minimum of 7.05 × 10^6^ CFU/100 mL to a maximum of 9.95 × 10^7^ CFU/100 mL and was reduced to below the detection limit (< 1 CFU/mL) after 6 hrs of disinfection (Table 4). *Escherichia coli* were also detected in all the untreated water samples (6 hrs of treatment) with counts ranging from a minimum of 2 CFU/100 mL to a maximum of 4 CFU/100 mL. The *E. coli* counts were then also reduced to below the detection limit after 6 hrs of disinfection.

**Table 4:** Indicator counts for solar disinfected water samples collected after 6 hrs of treatment and the corresponding untreated water samples collected at various temperatures

Disinfected Temperature	Indicator	Untreated Water Sample (Ave. CFU/100 mL)	Treated Water Sample (Ave. CFU/100 mL) after 6 hrs	Reduction (%)
52 °C	*E. coli*	2	BDL	>99
	HPC	7.05 × 10^6^	BDL	>99
68 °C	*E. coli*	4	BDL	>99
	HPC	7.95 × 10^7^	BDL	>99
70 °C	*E. coli*	2	BDL	>99
	HPC	9.95 × 10^7^	BDL	>99
75 °C	*E. coli*	2	BDL	>99
	HPC	8.7 × 10^7^	BDL	>99
89 °C	*E. coli*	2	BDL	>99
	HPC	1.45 × 10^7^	BDL	>99

(Note: *BDL* below detection limit)

The HPC in all the untreated water samples corresponding to 8 hrs of SODIS treatment ranged from a minimum of 1.45 × 10^7^ CFU/100 mL to a maximum of 9.95 × 10^7^ CFU/100 mL and was reduced to below the detection limit (< 1 CFU/mL) after 8 hrs of disinfection (Table 5). *Escherichia coli* were also detected in all the untreated water samples (8 hrs of treatment) with counts ranging from a minimum of 2 CFU/100 mL to a maximum of 13 CFU/100 mL. The *E. coli* counts were then also reduced to below the detection limit (< 1 CFU/mL) after 8 hrs of disinfection.

**Table 5:** Indicator counts for solar disinfected water samples collected after 8 hrs of treatment and the corresponding untreated water samples collected at various temperatures

Disinfected Temperature	Indicator	Untreated Water Sample (Ave. CFU/100 mL)	Treated Water Sample (Ave. CFU/100 mL) 8 hrs	Reduction (%)
63 °C	*E. coli*	4	BDL	>99
	HPC	7.95 × 10^7^	BDL	>99
67 °C	*E. coli*	2	BDL	>99
	HPC	9.95 × 10^7^	BDL	>99
72 °C	*E. coli*	13	BDL	>99
	HPC	9.4 × 10^7^	BDL	>99
76 °C	*E. coli*	2	BDL	>99
	HPC	8.7 × 10^7^	BDL	>99
86 °C	*E. coli*	2	BDL	>99
	HPC	1.45 × 10^7^	BDL	>99

(Note: *BDL* below detection limit)

For the untreated rainwater samples (6 hrs and 8 hrs), both the HPC and *E. coli* counts exceeded the drinking water guidelines stipulated by the DWAF [7]. However, after both 6 hrs and 8 hrs of SODIS treatment a significant (*p* < 0.05) reduction (>99%) in indicator counts was observed and all counts were within the DWAF [7] guidelines.

### Quantitative PCR for the detection of *Legionella* spp.

#### Quantitative PCR for the detection of viable *Legionella* spp. in SOPAS samples

**Fig. 2:**
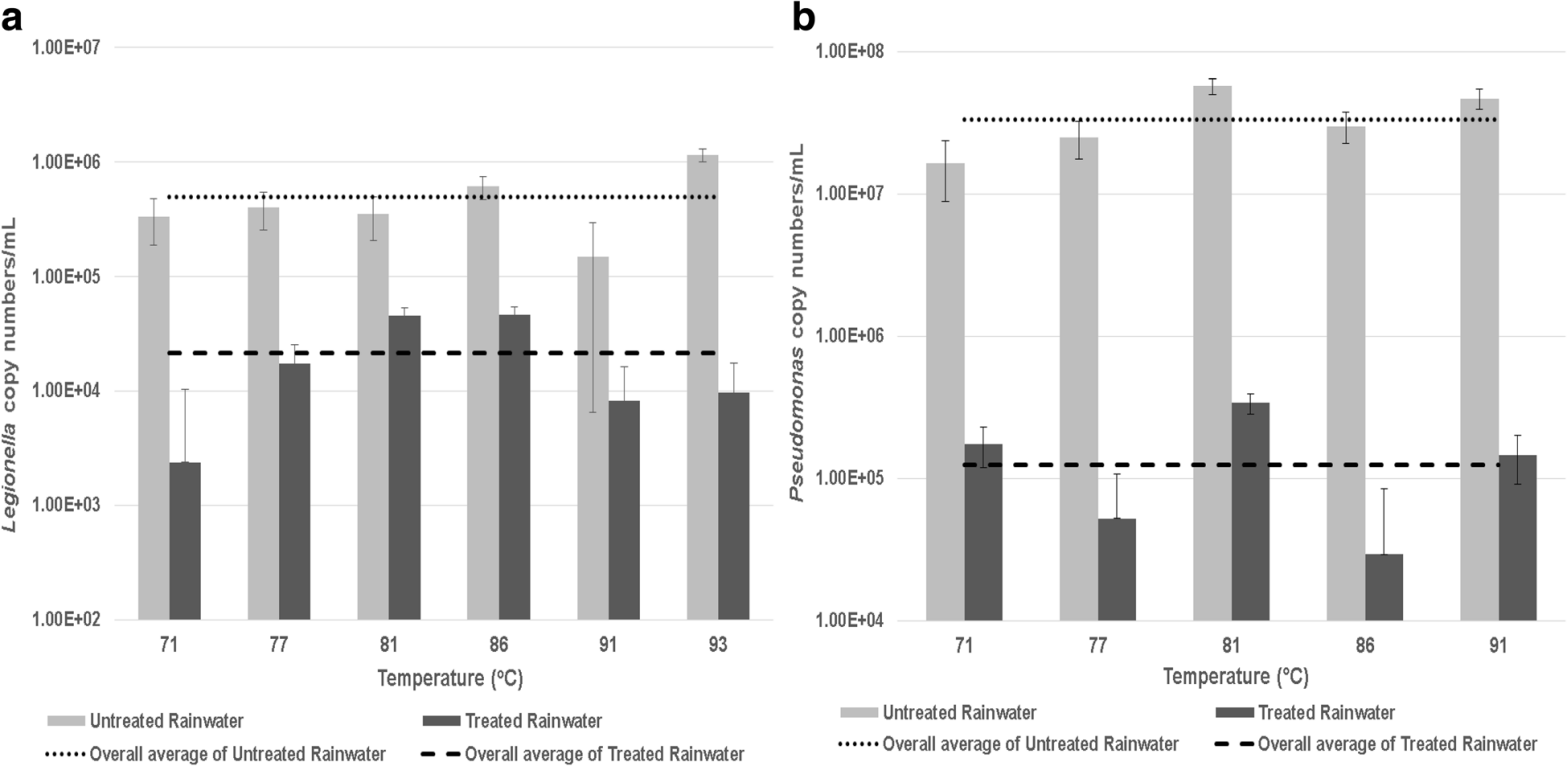
Viable (**a**) *Legionella* spp. and (**b**) *Pseudomonas* spp. gene copy numbers recorded in corresponding untreated and solar pasteurized rainwater samples collected at various temperatures. The overall average of *Legionella* and *Pseudomonas* spp. gene copy numbers of the untreated rainwater samples is indicated by a *dotted line*, while the overall average of *Legionella* and *Pseudomonas* spp. gene copy numbers of the treated rainwater samples is indicated by a *dashed line*. Error bar: SE (1SD) of duplicate samples analysed

The presence of viable *Legionella* cells in the untreated and corresponding treated SOPAS samples were determined using qPCR assays in conjunction with the EMA pre-treatment. A standard curve was constructed with a linear range of quantification from 10^8^ to 10^1^ gene copies per µL using the LightCycler®96 software Ver. 1.1.0.1320 (Roche Diagnostics International Ltd). A qPCR efficiency of 1.86 (93%) was obtained, with a linear regression coefficient (*R* ^*2*^) value of 0.98. Using the standard curve, viable *Legionella* copy numbers were quantified in the untreated and corresponding solar pasteurized water samples collected at various temperatures and are represented as 23S rRNA gene copies per mL (Fig. 2a).

A significant reduction (*p* < 0.05) in viable *Legionella* copy numbers after solar pasteurization of the rainwater samples collected at all temperature ranges (70 to 79 °C, 80 to 89 °C and 90 °C and above) was obtained (Fig. 2a). For the temperature range of 70 to 79 °C, an average of 1.74 × 10^5^ copies/mL was observed for the untreated water samples, which decreased to an average of 6.15 × 10^3^ copies/mL for the pasteurized water samples. For the temperatures ranging from 80 to 89 °C, an average of 4.79 × 10^5^ copies/mL was observed for the untreated water samples, compared to an average of 4.57 × 10^4^ copies/mL obtained for the pasteurized water. Lastly, for the temperatures 90 °C and above, an average of 6.49 × 10^5^ copies/mL for the untreated water samples was obtained, which decreased to an average of 8.92 × 10^3^ copies/mL for the pasteurized water samples.

At the lowest (70 to 79 °C) and highest (90 °C and above) pasteurization temperature ranges, a percentage reduction of 99.97% and 96.83% was observed (2-log reduction) in *Legionella* copy numbers, respectively, while the lowest percentage reduction (89.76%) in copy numbers was observed for the 80 to 89 °C temperature range (1-log reduction).

#### Quantitative PCR for the detection of viable *Legionella* spp. in SODIS samples

The same standard curve as described for the quantification of Pseudomonas copy numbers in th e untreated and SOPAS treated samples, was utilised to quantify viable *Legionella* copy numbers per mL for the untreated and corresponding solar disinfected water samples after 6 and 8 hrs (various temperatures recorded), respectively.

**Fig. 3:**
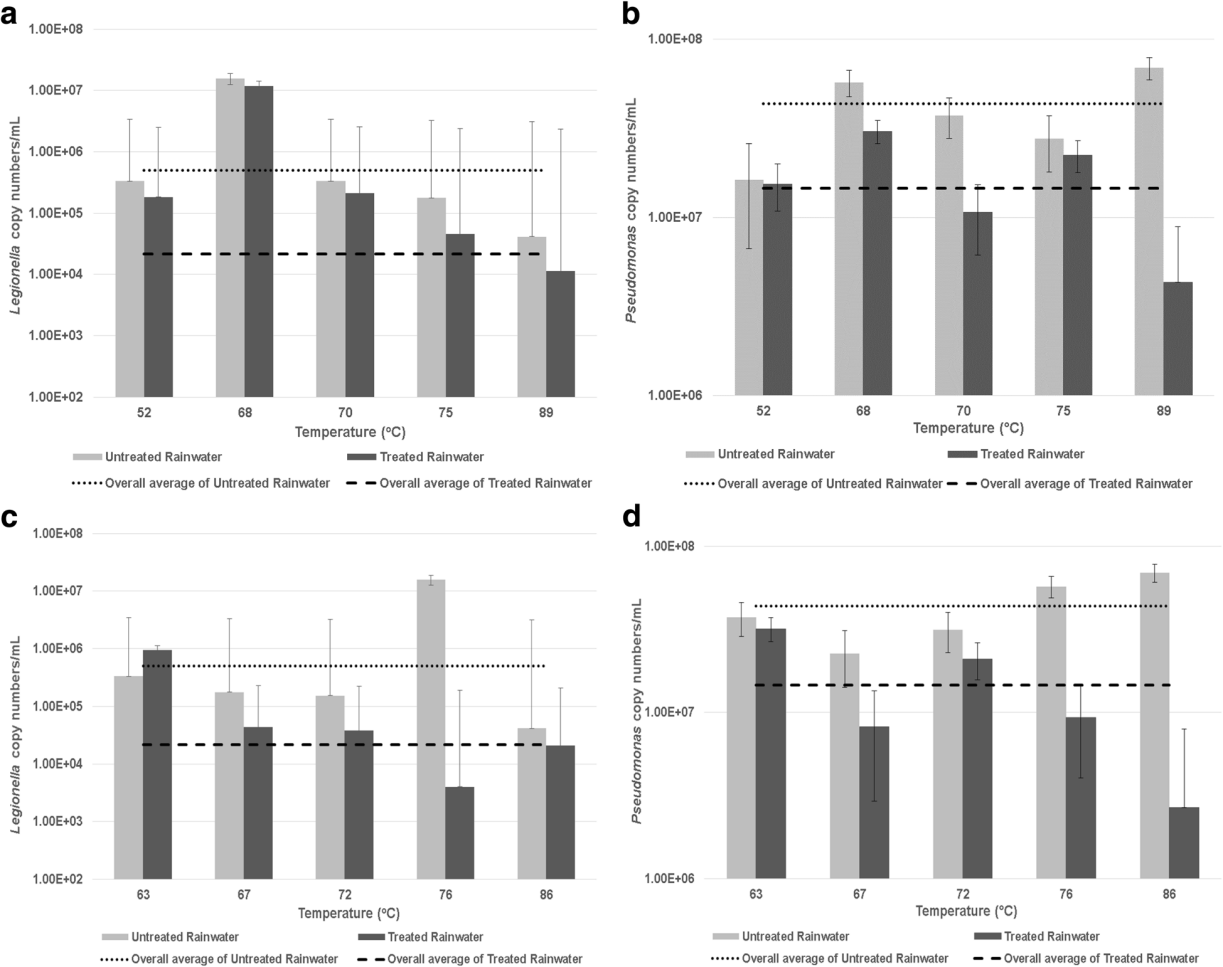
Viable (**a**) *Legionella* spp. and (**b**) *Pseudomonas* spp. gene copy numbers recorded in corresponding untreated and solar disinfected (for 6 hrs) rainwater samples collected at various temperatures. Viable (**c**) *Legionella* spp. and (**d**) *Pseudomonas* spp. gene copy numbers recorded in corresponding untreated and solar disinfected (for 8 hrs) rainwater samples collected at various temperatures. The overall average of *Legionella* and *Pseudomonas* spp. gene copy numbers of the untreated rainwater samples is indicated by a *dotted line*, while the overall average of *Legionella* and *Pseudomonas* spp. gene copy numbers of the treated rainwater samples is indicated by a *dashed line*. Error bar: SE (1SD) of duplicate samples analysed

The results obtained for the qPCR assays showed that there was a reduction in viable *Legionella* copy numbers after SODIS treatment for 6 hrs (Fig. 3a). The lowest percentage reduction (24.46%) in *Legionella* copy numbers was observed for a solar disinfected sample with a temperature of 68 °C, where *Legionella* copy numbers decreased from 1.56 × 10^7^ copies/mL for the untreated sample to 1.18 × 10^7^ copies/mL for the solar disinfected sample. The highest percentage reduction (74.09%) in copy numbers was observed for a solar disinfected sample with a temperature of 75 °C, where *Legionella* copy numbers decreased from 1.76 × 10^5^ copies/mL for the untreated sample to 4.56 × 10^4^ copies/mL for the solar disinfected sample. A significant (*p* < 0.05) reduction (72.6%) in *Legionella* copy numbers was also observed at 89 °C, where *Legionella* copy numbers of 4.13 × 10^4^copies/mL were observed for the untreated sample and then decreased to 1.13 × 10^4^ copies/mL after SODIS at 6 hrs.

The results obtained for the qPCR assays, indicated that overall there was a 2-log reduction in viable *Legionella* copy numbers (except 63 °C sample) after SODIS of 8 hrs for the rainwater samples collected at temperatures ranging from 67 to 86 °C (Fig. 3c). The lowest percentage reduction (50.07%) in copy numbers was observed for a SODIS temperature of 86 °C, where 4.12 × 10^4^ copies/mL was observed in the untreated sample compared to the solar disinfected sample where 2.06 × 10^4^ copies/mL was recorded. The highest percentage reduction (99.97%) in copy numbers was observed for a solar disinfected temperature of 76 °C, where 1.56 × 10^7^ copies/mL was observed in the untreated sample compared to the solar disinfected sample where 4.03 × 10^3^ copies/mL was recorded. For the temperatures of 67 °C and 72 °C, a percentage reduction in copy numbers of 75.43% and 75.41% was recorded, respectively. However, an increase in *Legionella* spp. copy numbers was observed for the solar disinfected sample with a temperature of 63 °C, where 3.32 × 10^5^copies/mL was observed in the untreated sample compared to 9.54 × 10^5^ copies/mL recorded in the solar disinfected sample.

### Quantitative PCR for the detection of *Pseudomonas* spp.

#### Quantitative PCR for the detection of viable *Pseudomonas* spp. in SOPAS samples

The quantification of viable *Pseudomonas* cells in the untreated and corresponding treated SOPAS samples was determined using qPCR assays in conjunction with the EMA pre-treatment. A standard curve was constructed with a linear range of quantification from 10^9^ to 10^0^ gene copies per μL using the software LightCycler®96 Version 1.1.0.1320 (Roche Diagnostics International Ltd). A qPCR efficiency of 1.83 (92%) was obtained, with a linear regression coefficient (*R* ^*2*^) value of 1.00. Using the standard curve, viable *Pseudomonas* copy numbers were quantified in the untreated and corresponding solar pasteurized (treated) water samples collected at various temperatures and are represented as *Pseudomonas* lipoprotein *oprI* gene copies per mL (Fig. 2b).

A significant reduction (*p* < 0.05) in viable *Pseudomonas* copy numbers after solar pasteurization of the rainwater samples collected at all temperature ranges (70 to 79 °C, 80 to 89 °C and 90 °C and above) was obtained (Fig. 2b). For the temperature range of 70 to 79 °C, an average of 2.07 × 10^7^ copies/mL was observed for the untreated water samples, which decreased to an average of 1.13 × 10^5^ copies/mL for the pasteurized water samples. For the temperatures ranging from 80 to 89 °C, an average of 4.37 × 10^7^ copies/mL was observed for the untreated water samples, compared to an average of 1.84 × 10^5^ copies/mL obtained for the pasteurized water. Lastly, for the temperatures ranging from 90 °C and above, an average of 3.57 × 10^7^ copies/mL for the untreated water samples was obtained, which decreased to an average of 7.31 × 10^4^ copies/mL for the pasteurized water samples. It should however be noted that while an average of 2.45 × 10^7^ *Pseudomonas* copies/mL was observed in the untreated water sample (collected with the 93 °C SOPAS sample), no amplification of the *oprI* gene was recorded in the 93 °C pasteurized water sample resulting in a C_q_ value below detection limit obtained (not presented on Fig. 2b).

For the pasteurization temperature ranges of 70 to 79 °C and 80 to 89 °C a reduction of 99.45% and 99.58% was observed in *Pseudomonas* copy numbers, respectively, thus a 2-log reduction was observed for both these temperature ranges. In addition, the greatest percentage reduction of 99.80% (3-log reduction) in copy numbers was observed for the 90 °C and above temperature range.

#### Quantitative PCR for the detection of viable *Pseudomonas* spp. in SODIS samples

The same standard curve as described for the quantification of Pseudomonas copy numbers in th e untreated and SOPAS treated samples, was utilised to quantify viable *Pseudomonas* copy numbers per mL for the untreated and corresponding solar disinfected water samples after 6 and 8 hrs (various temperatures recorded), respectively.

The results obtained for the qPCR assays showed that there was a reduction in viable *Pseudomonas* copy numbers after SODIS treatment for 6 hrs (Fig. 3b). The lowest percentage reduction (5.53%) in *Pseudomonas* copy numbers was observed for a solar disinfected sample with a temperature of 52 °C, where *Pseudomonas* copy numbers decreased from 1.63 × 10^7^ copies/mL for the untreated sample to 1.54 × 10^7^ copies/mL for the solar disinfected sample. The highest percentage reduction (93.73%) in copy numbers was observed for a solar disinfected sample with a temperature of 89 °C, where *Pseudomonas* copy numbers decreased from 6.90 × 10^7^ copies/mL for the untreated sample to 4.33 × 10^6^ copies/mL for the solar disinfected sample yielding a 1-log reduction.

The results obtained for the qPCR assays, indicated that there was an overall 1-log reduction in viable *Pseudomonas* copy numbers after SODIS of 8 hrs (Fig. 3d) for the rainwater samples collected at temperatures ranging from 63 to 86 °C. The lowest percentage reduction (14.37%) in copy numbers was observed for a SODIS temperature of 63 °C, where 3.73 × 10^7^ copies/mL was observed in the untreated sample compared to the solar disinfected sample where 3.19 × 10^7^ copies/mL was recorded. The highest percentage reduction (96.12%) in copy numbers was observed for a solar disinfected temperature of 86 °C, where 6.90 × 10^7^ copies/mL was observed in the untreated sample compared to the solar disinfected sample where 2.68 × 10^6^ copies/mL was recorded.

## Discussion

The efficiency of two solar based treatment systems (SOPAS and SODIS) were evaluated for the treatment of roof harvested rainwater. Numerous chemical and microbial parameters were investigated in order to determine which system effectively improved the overall quality of the harvested rainwater to within drinking water guidelines. Chemical analysis of the solar pasteurized and corresponding untreated rainwater samples then indicated that all cation (with the exception of Pb and Ni) and anion concentrations were within the drinking water guidelines as stipulated by the ADWG [37], DWAF [7] and SANS 241 [38]. Nickel and Pb were detected in all three pasteurization water samples (71 °C, 86 °C and 93 °C) analysed at concentrations exceeding the drinking water guidelines. Although the SOPAS system has a storage tank constructed from high grade polyethylene, it contains SABS approved Ni coated dezincification resistant (DZR) brass connector points utilised for mounting purposes. Nickel could have thus leached from the Ni coated brass metal during exposure to high temperatures in the SOPAS system. However, only long term exposure to Ni at high concentrations may be toxic to humans as the concentration of beta-microglobulin increases in the kidneys [37]. In addition, the Pb detected could have leached from the surface of the polyethylene storage tank into the water, as the high grade polyethylene storage tank is treated with Pb (personal communication, Crest Organization) which acts as a stabilizer and is often used to treat polyethylene surfaces exposed to high temperature [39]. Significantly high concentrations of Pb have a severe effect on the human central nervous system and results in the interference with calcium metabolism (bone formation), red blood cell production and contributes to kidney failure [37].

For the SODIS system, chemical analysis revealed that the cation (with the exception of Fe) and anion concentrations, were also within the drinking water guidelines as stipulated by the ADWG [37], DWAF [7] and SANS 241 [38]. It should however, be noted that the untreated water samples had iron concentrations which exceeded the drinking water guidelines. These concentrations then increased in the SODIS samples treated for 6 and 8 hrs, respectively. Suib [40] indicated that the synergistic effect of solar photons and hydrogen peroxide generates hydroxide inside microbial cells by Fenton’s reaction, causing Fe and hydrogen peroxide to flow through the cell membrane. Furthermore, when cells are irradiated with near UV photons, an increase in ferrous (Fe^2+^) iron occurs due to increased membrane permeability, resulting in an increased Fe concentration in the surrounding environment. As SODIS uses both heat and UV to treat the water samples, this phenomenon could have been observed in the treated water samples.

Numerous studies have indicated that the microbial quality of harvested rainwater does not comply with drinking water guidelines [18, 41, 42]. The untreated rainwater, SOPAS and SODIS rainwater samples were thus analysed for the presence of the indicator bacteria *E. coli*, HPC, enterococci and faecal coliforms. *Escherichia coli* and HPC were detected in all the untreated water samples collected for SOPAS analysis, and were effectively reduced (>99%) to below the detection limit in all the samples collected at the various temperature ranges (71 °C to 93 °C). These results correlate with a study conducted by Dobrowsky et al. [17], where the research group showed that indicator counts in solar pasteurized water were reduced to below the detection level at temperatures of 72 °C and above. Similar to the results obtained for the SOPAS system, the *E. coli* and HPC counts recorded in the untreated water samples were also above the drinking water guidelines as stipulated by DWAF [7] and were reduced to below the detection limit after 6 and 8 hrs of SODIS treatment, with a minimum final temperature of 52 °C and 63 °C recorded, respectively. A study conducted by Berney et al. [43] showed that SODIS with strong irradiation conditions of up to 6 hrs disrupts a sequence of basic cellular functions in *E. coli* that leads to cell death. Overall the results thus indicate that the SOPAS system and SODIS systems (6 and 8 hrs of treatment), successfully reduced indicator bacteria numbers by >99%, at a minimum temperature of 71 °C for the SOPAS system and 52 °C for the SODIS system. These results correlate to a study conducted by Spinks et al. [18] where the research group suggested that a minimum temperature of 55 °C was sufficient to eliminate enteric pathogenic bacteria in water samples.

A poor correlation between indicator microorganisms and opportunistic bacteria has however, been reported [44–46] as previous studies have shown that opportunistic bacteria, such as *Legionella* and *Pseudomonas* spp. amongst others, persist in roof harvested rainwater when low indicator counts are recorded [17, 42]. Oliver [47] then indicated that opportunistic pathogenic bacteria such as *Legionella* spp. are able to enter a viable but non-culturable state and therefore in the current study, EMA-qPCR assays were utilised to test for the presence and viability of these organisms in solar pasteurized and solar disinfected treated rainwater samples. Although conventional PCR can effectively be utilised as a presence/absence indicator of a particular gene or organism, it cannot be used to indicate the viability of the organism detected. In contrast, EMA-qPCR can be used to analyse for the presence and the viability of an organism and is considered a beneficial method for the detection and quantification of intact microorganisms [20, 48].

The EMA-qPCR assays indicated that a significant (*p* < 0.05) reduction (94.70%) in viable *Legionella* copy numbers was obtained after SOPAS and yielded a 2-log reduction overall. For the SODIS system, *Legionella* copy numbers also decreased in samples treated for 6 and 8 hrs, respectively. In addition, treatment after 8 hrs yielded a greater decrease (75.22%) in copy numbers (2-log reduction) in comparison to treatment for 6 hrs (maximum of 1-log reduction for the various temperatures), where a 50.60% reduction was observed. However, an increase in copy numbers was obtained for one solar disinfection sample (63 °C) treated for 8 hrs. It is known that *Legionella* can form associations with protozoa where they exist as intracellular parasites and are able to proliferate at temperatures from 50 °C to 65 °C due to the presence of heat shock proteins [49, 50]. *Legionella* spp. are therefore able to out compete other organisms and survive at these high temperatures (>90 °C) [51]. Moreover, a study conducted by Vervaeren et al. [50] showed that *L. pneumophila* is able to proliferate in heat treated water (up to a temperature of 70 °C). According to a study conducted by Hussong et al. [52] viable but non-culturable *Legionella* spp. also regain culturability and remain pathogenic when favourable conditions arise.

The EMA-qPCR assays for *Pseudomonas* yielded similar results to those obtained for *Legionella*. A reduction of 99.61% (3-log reduction) in viable *Pseudomonas* copy numbers was obtained after SOPAS treatment. In addition, SODIS treatment after 8 hrs yielded a greater reduction of 58.31% in viable copy numbers of *Pseudomonas* spp. in comparison to treatment for 6 hrs (47.27%). It is hypothesized that samples treated for 8 hrs were exposed to UV irradiation for an extended time period resulting in a greater microbial reduction. Furthermore, it is well known that *Pseudomonas* can enter a viable but non-culturable state [53] and results obtained in the current study indicated that *Pseudomonas* spp. remain viable at a temperature of 89 °C after treatment by SODIS. Several studies [54–56] have thus utilised the addition of a photocatalytic material, to enhance the effect of microbial inactivation over a wide range of microorganisms and thereby increase the efficiency of a SODIS system. Titanium dioxide (TiO_2_) is considered the most suitable photocatalyst due to the lack of toxicity and chemical and photochemical stability, however further research is needed to determine to potability of TiO_2_ treated water [57].

Results obtained in the current study indicated that SOPAS treatment yielded a greater reduction in viable *Legionella* and *Pseudomonas* spp. (94.70% and 99.61%, respectively) copy numbers, compared to SODIS treatment after 6 (50.60% for *Legionella* spp. and 47.27% for *Pseudomonas* spp.) and 8 hrs (75.22% for *Legionella* spp. and 58.31% for *Pseudomonas* spp.). While not significant, treatment with SOPAS yielded a lower reduction in viable *Legionella* copy numbers compared to *Pseudomonas* copy numbers. It is hypothesized that *Legionella* spp. may have been able to persist due to: the presence of heat shock proteins to protect them from high temperatures; associations with amoebae species; and the formation of biofilms [58].

## Conclusions and future research

Based on the indicator count analysis, treatment of harvested rainwater with both SOPAS and SODIS improved the microbial quality of rainwater and the water could be utilised for irrigation and domestic purposes such as cooking, laundry and washing. The SOPAS system can however, effectively treat larger volumes of rainwater in comparison to the SODIS system and based on the EMA-qPCR results obtained in the current study, SOPAS was the most effective for the reduction of viable *Legionella* and *Pseudomonas* spp. copy numbers in harvested rainwater. However, depending on the material utilised to construct the storage tank, metals and chemicals may leach into the water when temperatures higher than 71 °C are achieved inside the SOPAS system. In contrast, SODIS systems function as batch culture systems and are more cost-effective and easier to operate and maintain. Future research should however, focus on up-scaling SODIS systems to allow for the efficient treatment of larger volumes of rainwater.

## Abbreviations

ANOVA: Analysis of variance
Ave.: Average
BDL: Below detection limit
bp: Base pairs
CAF: Central analytical facility
CFU: Colony forming units
C_q_: Quantitative cycle
DNA: Deoxyribonucleic acid
DWAF: Department of Water Affairs and Forestry
DZR: Dezincification resistant
EMA: Ethidium monoazide
EMA-qPCR: Ethidium monoazide bromide quantitative polymerase chain reaction
HPC: Heterotrophic plate count
Hrs: Hours
ICP-AES: Inductively coupled plasma atomic emission spectrometry
m-FC: Membrane filter faecal coliform
MLGA: Membrane lactose glucuronide agar
NTU: Nephelometric turbidity unit
PCR: Polymerase chain reaction
PET: Polyethylene terephthalate
qPCR: Quantitative PCR
*R*^2^: Linear regression coefficient
R2A: Reasoner’s 2A agar
rRNA: Ribosomal ribonucleic acid
RWH: Rainwater harvesting
SANS: South African National Standards
SD: Standard deviation
SODIS: Solar disinfection
SOPAS: Solar pasteurization
USEPA: United States Environmental Protection Agency
UV: Ultra-violet
WHO: World Health Organization
